# Amperometric Sensing of Carbon Monoxide: Improved Sensitivity and Selectivity via Nanostructure-Controlled Electrodeposition of Gold

**DOI:** 10.3390/bios11090334

**Published:** 2021-09-13

**Authors:** Taehui Kwon, Hee Young Mun, Sunghwa Seo, Areum Yu, Chongmok Lee, Youngmi Lee

**Affiliations:** Department of Chemistry & Nanoscience, Ewha Womans University, Seoul 03760, Korea; kwonth9601@gmail.com (T.K.); 1993yhm@naver.com (H.Y.M.); ssh5462@naver.com (S.S.); aremyou@ewha.ac.kr (A.Y.); cmlee@ewha.ac.kr (C.L.)

**Keywords:** gold, electrodeposition, surface hydrophobicity, carbon monoxide, amperometric sensing

## Abstract

A series of gold (Au) nanostructures, having different morphologies, were fabricated for amperometric selective detection of carbon monoxide (CO), a biologically important signaling molecule. Au layers were electrodeposited from a precursor solution of 7 mM HAuCl_4_ with a constant deposition charge (0.04 C) at various deposition potentials. The obtained Au nanostructures became rougher and spikier as the deposition potential lowered from 0.45 V to 0.05 V (vs. Ag/AgCl). As prepared Au layers showed different hydrophobicity: The sharper morphology, the greater hydrophobicity. The Au deposit formed at 0.05 V had the sharpest shape and the greatest surface hydrophobicity. The sensitivity of an Au deposit for amperometric CO sensing was enhanced as the Au surface exhibits higher hydrophobicity. In fact, CO selectivity over common electroactive biological interferents (L-ascorbic acid, 4-acetamidophenol, 4-aminobutyric acid and nitrite) was improved eminently once the Au deposit became more hydrophobic. The most hydrophobic Au was also confirmed to sense CO exclusively without responding to nitric oxide, another similar gas signaling molecule, in contrast to a hydrophobic platinum (Pt) counterpart. This study presents a feasible strategy to enhance the sensitivity and selectivity for amperometric CO sensing via the fine control of Au electrode nanostructures.

## 1. Introduction

Carbon monoxide (CO) is generally considered to be a toxic pollutant, but its endogenous production at low levels is essential for mammals due to its important physiological roles as a signaling molecule mediating vasodilation, platelet aggregation, neurotransmission, and neuromodulation [1,2]. Thus, diverse methods for the measurement of CO have been developed for environmental and biological applications. The usually used methods for CO measurements are gas chromatography [3], mass spectrometry [4], luminescence [5], fluorescence [6], colorimetry [7], and electrochemical sensors [8]. Although each of these techniques has its own strengths and weaknesses, electrochemical methods are advantageous particularly for the real-time direct measurement of CO in a confined space. In fact, electrochemical sensors can monitor CO levels quantitatively with fast response time and high sensitivity/selectivity [9,10]. 

In electrochemical CO sensors operating in amperometric mode, CO oxidation current is generally measured as a signal proportional to CO concentration while a constant potential (to cause CO oxidation) is applied to a working electrode of the sensor. Thus, any co-present species, which can undergo electrode reaction at the selected working electrode potential, intrude into the measured current. Common electroactive biological interferents are nitrite, acetaminophen, ascorbic acid, etc. To improve the sensor selectivity to CO over these interferents, polymer membranes such as Nafion/chitosan [11] and polytetrafluoroethylene [12] are usually employed on the working electrode surface. However, the additional introduction of membranes makes negative aspects of the sensors (e.g., more complicated fabrication process and slower response time due to the hindered CO transport through the membrane).

The synthesis of metallic nanostructures has been studied by many researchers because these materials are applicable to various fields such as sensors [13,14], electrocatalysis [15], heterogeneous catalysis [16], and fuel cells [17]. The surface properties of these nanostructures can be changed with the variation of the types/compositions of metals, dimensions, and shapes [18,19]. Among the assorted techniques used for synthesizing metal nanostructures (e.g., vapor deposition, wet synthesis, template supported synthesis, etc. [19,20]), electrodeposition is the most straightforward method to build nanostructures directly on the electrode surface with the morphology control [21,22,23]. Electrodeposited nanostructures having various shapes such as nano-trees [22], dendrites [24], and spikes [25] have been prepared with the regulation of the composition/concentration of a precursor solution and charge/potential applied for the deposition, etc.

The wettability of a solid surface is significantly reduced by making the surface rougher [26]. Thus, nanostructured surfaces have different wettability relying on the actual morphology. For instance, the superhydrophobic properties of flowerlike Au structures [27] and Pd nanoflakes [23] synthesized with electrodeposition have been reported. Since most common biological interferents are polar or ionic species, hydrophobically a nanostructured electrode surface is anticipated to exhibit a selectivity to nonpolar/neutral species. In fact, we previously prepared greatly hydrophobic sharp-pointed Pt nanostructures via electrodeposition and applied them for membrane-free amperometric sensing of nitric oxide (NO), another physiologically important signaling gas molecule [22]. In this current study, various Au nanostructures with different morphologies and hydrophobicity are fabricated by simply changing the applied potential via electrodeposition method. Furthermore, as-prepared Au nanostructures are investigated regarding their feasibility as amperometric CO sensors (e.g., sensitivity and selectivity to CO) depending on the morphology and hydrophobicity for the first time.

## 2. Materials and Methods

### 2.1. Chemicals and Materials

Gold(III) chloride trihydrate (HAuCl_4_·3H_2_O), lead(II) acetate trihydrate (Pb(CH_3_COO)_2_·3H_2_O), potassium tetrachloroplatinate(II) (K_2_PtCl_4_), sulfuric acid (H_2_SO_4_), sodium nitrite (NaNO_2_), 4-acetamidophenol (AP), L-ascorbic acid (AA), and nafion perfluorinated resin solution were from Sigma-Aldrich (St. Louis, MO, USA). 4-Aminobutyric acid (GABA) was from Alfa Aesar (Heysham, England). Phosphate-buffered saline (PBS, pH 7.4 ± 0.1 at 25 °C) was from Welgene Inc. (Korea). Argon (Ar), nitric oxide (NO), and carbon monoxide (CO) gases were from Dong-A Gas Co. (Korea). All aqueous solutions were prepared with deionized water (resistivity ≥ 18 MΩ·cm), and all chemical compounds were analytical grade and used without further purification.

### 2.2. Electrodeposition of Au Structures

As substrates for electrodeposition, Au disk electrodes (Bioanalytical Systems, Inc., West Lafayette, IN, USA, 2 mm in diameter) were used. An Ag/AgCl (CH Instruments, Inc., Austin, TX, USA) and an Au wire (Sigma-Aldrich, St. Louis, MO, USA, 1 mm in diameter) was used as the reference and counter electrode, respectively.

Before the electrodeposition of Au nanostructures, Au disk substrate electrodes were wet-polished on a polishing cloth with 0.3-µm alumina powder. To remove the alumina slurry, the electrodes were sonicated in deionized water for 10 min. A deposition solution was an aqueous solution containing 7 mM HAuCl_4_ and 0.1 mM Pb(CH_3_COO)_2_ that was purged using Ar gas for 10 min before deposition. Au nanostructured layers were electrodeposited on the Au disk electrodes in the prepared solution with amperometry up to the deposition charge of 0.04 C at various applied potentials: 0.05, 0.15, 0.20, 0.25 and 0.45 V (vs. Ag/AgCl). A CHI 900B bipotentiostat (CH Instruments, Inc., Austin, TX, USA) was used for electrodeposition.

### 2.3. Physical and Electrochemical Characterization

The physical characterization of electrodeposited Au surfaces was performed using field emission scanning electron microscopy (FE-SEM, Jeol JSM-6700F, Tokyo, Japan) equipped with an energy dispersive X-ray spectrometer (EDS). The water contact angles were measured by ImageJ (Image Processing and Analysis in Java) with a droplet of 1 µL deionized water on the electrodeposited Au electrode. 

All electrochemical processes were conducted using the same electrodes and potentiostat described in the electrodeposition procedure. Linear sweep voltammetry (LSV) was performed in PBS solution (pH 7.4) containing 50 µM CO, NO or AA. For the amperometric measurements, the electrodes were polarized in a PBS solution (pH 7.4) at −0.05 V (vs. Ag/AgCl) until steady-state currents maintained. Then, current responses of the electrodes were observed while an aliquot of saturated CO (0.9 mM) or NO (1.91 mM) stock solutions was successively injected to a gas-tight cell containing deaerated PBS solution being magnetically stirred. The CO and NO stock solutions were prepared by bubbling CO and NO gases individually in deaerated PBS solutions [12,22]. The electrode current responses to common biological interfering species (e.g., nitrite, AP, GABA, and AA) were also measured. To measure the real surface areas (RSAs) of electrodeposited Au electrodes, cyclic voltammetry (CV) was performed in 0.05 M H_2_SO_4_ aqueous solution with a scan rate of 100 mV·s^−1^. The electrode RSA was estimated from the integrated area of Au oxide layer reduction peak in the obtained CV curve using a conversion factor (386 µC·cm^−2^) [28]. 

## 3. Results and Discussion

### 3.1. Au Nanostructures Depending on the Deposition Potentials

**Figure 1:**
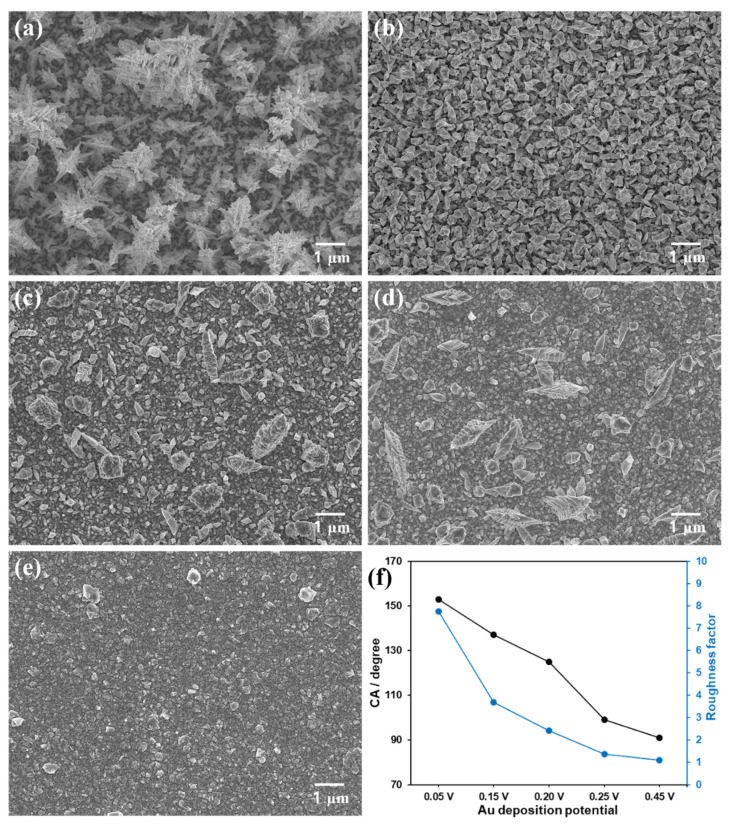
(**a**–**e**) SEM images of the Au nanostructures electrodeposited with various applied potentials of (**a**) 0.05 V, (**b**) 0.15 V, (**c**) 0.20 V, (**d**) 0.25 V and (**e**) 0.45 V vs. Ag/AgCl. (**f**) Static water contact angles and roughness factors of the Au surfaces as a function of the deposition potential (*n* = 5).

For the electrodeposition of Au layers on flat Au disk substrates, the same precursor solution (7 mM HAuCl_4_ with a seeding material of 0.1 mM Pb(CH_3_COO)_2_ in water) and the constant deposition charge (0.04 C) were employed. The variation of only the electrode potential applied for the deposition successfully changed the actual morphology of the deposited Au nanostructures. A much sharper morphology was formed as the deposition potential was lowered from 0.45 V to 0.05 V (Figure 1a–e). The Au deposit prepared at 0.05 V showed a very sharp pine tree-like structure and grew the highest in height from the substrate as shown in Figure 1a. In the Au structure with a deposition potential of 0.15 V, less sharp shapes grew to a lower height than that with 0.05 V (Figure 1b). Both of the Au structures deposited at 0.20 and 0.25 V had similarly grown shapes of some spiky nanostructures on dense and relatively smooth bottom layers. In more detail, the deposit obtained at 0.20 V appeared to have a rougher bottom layer and a larger number of small spikes than that formed at 0.25 V (Figure 1c,d). The one prepared at 0.45 V had the smoothest morphology with evenly distributed round shaped nanostructures with nearly no upward grown ones. The more positive deposition potential was applied, the less sharp morphology was obtained presumably due to a slower cathodic deposition rate [29]. As can be seen in Figure S1, the more positive deposition potential required the longer time to reach the same deposition charge of 0.04 C, indicating the slower deposition rate. Under the condition of a slow electrodeposition, Au precursor ions have a chance to access within the inner pores of the pre-deposited Au layer and then are reduced within those pores, producing densely packed nanostructures. In the case of the least positive deposition potential allowing the faster deposition speed, Au precursor ions are mainly reduced at the outermost parts of the pre-deposited Au layer, producing sharp spiky structures. Meanwhile, the Au layer deposited from a solution containing only HAuCl_4_ had a much less sharp morphology (Figure S2). Thus, Pb(CH_3_COO)_2_ was necessary to produce a sharply nanostructured Au deposit. Pb^2+^ ions from Pb(CH_3_COO)_2_ are thought to play a role in directing the Au deposit growth pattern as previously reported [30].

Static water contact angles of the Au nanostructures deposited with different deposition potentials were investigated (Figure S3). The tendency of the water contact angle values is shown in Figure 1f as a function of the deposition potential. The Au deposit with a deposition potential of 0.05 V exhibited a contact angle of 153°, indicating a superhydrophobic property of the surface [31]. The contact angles of the Au surfaces decreased gradually down to ~90° as the deposition potential became more positive from 0.05 V to 0.45 V. This clearly presents that the surface with a sharper morphology exhibits more hydrophobic property, as previously reported for electrodeposited Pt nanostructures [21,22]. In fact, the Au deposit formed at the most negative potential had the sharpest morphology and accordingly the greatest hydrophobicity.

### 3.2. Electrochemical Characterization of Au Nanostructures

**Figure 2:**
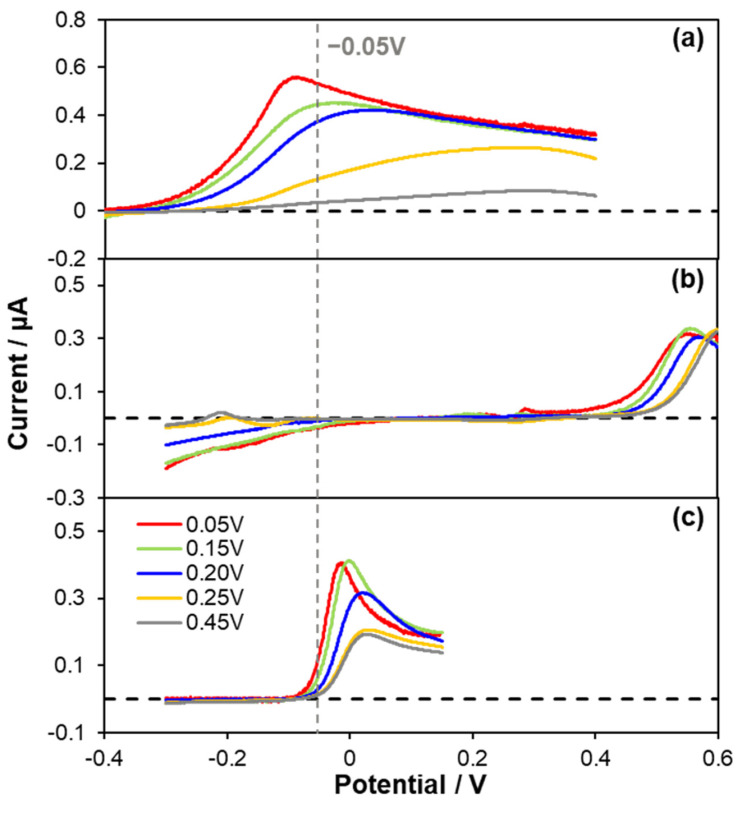
Background−corrected linear sweep voltammetry (LSV) curves of the Au electrodes deposited at various deposition potentials (0.05 V to 0.45 V) obtained in deaerated PBS solution (pH 7.4) containing 50 μM (**a**) CO, (**b**) NO and (**c**) AA with a scan rate of 10 mV·s^−1^. The dashed lines represent current zero lines.

In order to find an optimum applied potential for amperometric CO sensing, LSV was carried out for the oxidation of CO. In addition, the electrochemical reactions of NO (a similar electroactive gasotransmitter) and AA were investigated with LSV, because Au nanomaterials have also been reported to be good electrocatalysts for AA oxidation [32,33,34]. As presented in Figure 2, the oxidations of CO, NO and AA were observed at the various Au deposits within the experimental potential scan range. In Figure 2a, it is noticed that the Au electrodeposited at 0.05 V exhibits the least positive onset potential for CO oxidation, and CO oxidation occurred at more positive potential as the Au deposition potential became more positive. This tendency was the same for NO and AA oxidation while CO oxidation behavior was most significantly dependent of the deposited Au morphology. It implies that the sharp hydrophobic structure of Au is the most beneficial for CO oxidation. The LSV curves of NO and AA depending on Au deposits are possibly attributed to the difference of electrochemical surface area (ESA) of Au rather than the morphology effect. In fact, the Au layer deposited at less positive potential exhibited larger ESA (vide infra). Correspondingly, the roughness factors of the deposits were also increased as the deposition potential became less positive (Figure 1f).

**Figure 3:**
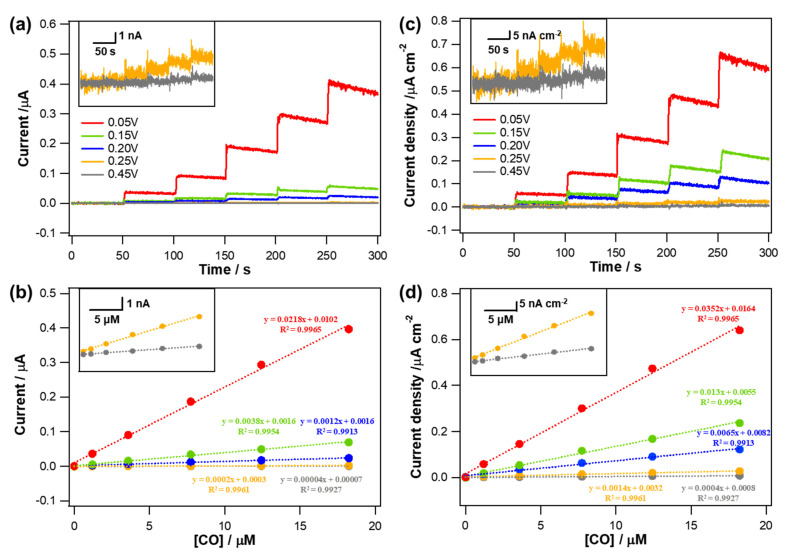
(**a**) Typical dynamic current response curves of the various Au electrodes electrodeposited at different deposition potentials (0.05 V to 0.45 V) to CO concentration changes (0~18.2 µM) in deaerated PBS solution (pH 7.4) measured at an applied potential of −0.05 V (vs. Ag/AgCl); (**b**) the corresponding calibration curves. Insets show graphs for the Au electrodes electrodeposited at 0.25 and 0.45 V with a magnified y−axis scale. (**c**,**d**) The current data in (**a**,**b**) were normalized to the corresponding electrode ESAs.

According to the LSV results shown in Figure 2, −0.05 V (vs. Ag/AgCl), where both NO reduction and AA oxidation were relatively negligible, was chosen as an electrode applied potential to monitor amperometric response to CO. Anodic currents induced by CO oxidation occurring at the Au electrodes applied with −0.05 V (vs. Ag/AgCl) were monitored while an aliquot of CO stock solution (0.9 mM) was added into a PBS solution successively to increase the CO concentration. Typical dynamic current response curves and corresponding calibration curves are shown in Figure 3a,b. All the Au electrodes exhibited the anodic currents increased in a good linear proportion to the increased CO concentration levels (*R*^2^ > 0.99) within a tested concentration range (0~18.2 µM). In consistent with the LSV results, the actual degree in the increased current responding to the same CO concentration increment was different depending on the type of Au deposit. The greater current response was observed for the sharper morphology and therefore the Au deposit formed at 0.05 V showed the highest current sensitivity to CO (*S_I_*_,CO_).

To rule out the effect caused by the different areas among the electrodes, the measured current was normalized to the ESA of each electrode, which was estimated using a conversion factor of 386 µC·cm^−2^ for Au oxide monolayer reduction [28]. The measured mean ESAs (±st. dev., *n* = 5) were 0.620 (±0.015), 0.294 (±0.046), 0.193 (±0.017), 0.109 (±0.015), and 0.086 (±0.005) cm^2^ for the Au structures formed with deposition potentials of 0.05, 0.15, 0.20, 0.25, and 0.45 V, respectively. As confirmed in the SEM images (Figure 1), Au deposit formed at more positive potential had smoother morphology. Accordingly, the ESA of the Au deposit decreased with increasing deposition potential because the roughness of the electrode diminished. Even after the normalization to the corresponding electrode ESA, the resulting current density sensitivity (*S_J_*_,CO_) showed a tendency consistent with *S_I_*_,CO_. The sharper morphology the Au deposit has, the higher *S_J_*_,CO_ it shows (Figure 3c,d). In fact, the both *S_I_*_,CO_ and *S_J_*_,CO_ of the Au deposits were decreased as the deposition potential became more positive. It is inferred that the different hydrophobicity of an Au deposit caused by the distinct nanostructure clearly affect the CO oxidation. As the hydrophobicity of the Au electrodes increased, CO oxidation was more effectively facilitated and therefore the sensitivity was increased.

The sensitivity was defined as the slope of a calibration curve (Figure 3b,d) and calculated from the following equation:

(1) $$S_{I,\mathrm{CO}}=\frac{I}{C_{\mathrm{CO}}}\;\mathrm{and}\;S_{J,\mathrm{CO}}=\frac{J}{C_{\mathrm{CO}}}$$

where *S_I_*_,CO_ and *S_J_*_,CO_ are the current sensitivity (µA·µM^−1^) and current density sensitivity (µA·cm^−2^·µM^−1^) to CO, respectively; *I* is the current (µA); *J* is the current density (µA·cm^−2^); and *C*_CO_ is the concentration of CO (µM).

**Figure 4:**
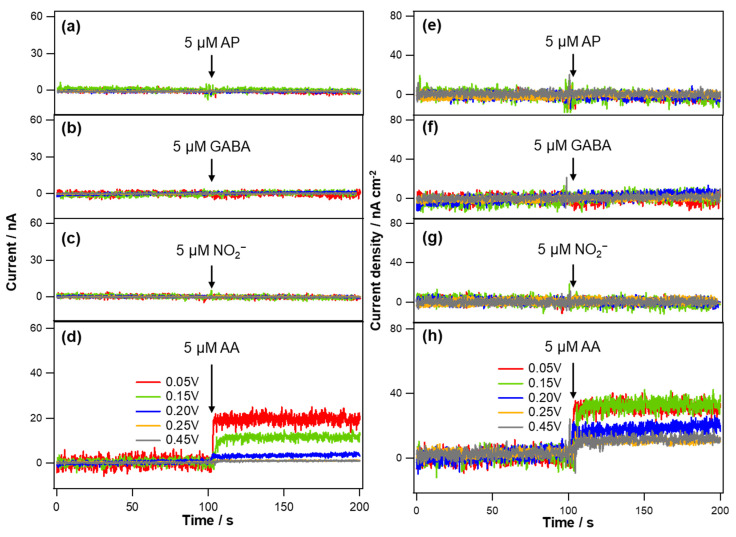
Typical current responses of the Au electrodes deposited with various deposition potentials to 5 µM of (**a**) AP, (**b**) GABA, (**c**) NO_2_^−^ and (**d**) AA in deareated PBS solution (pH 7.4) measured at an applied potential of −0.05 V (vs. Ag/AgCl). (**e**–**h**) The current data in (**a**–**d**) were normalized to the corresponding electrode ESAs.

**Figure 5:**
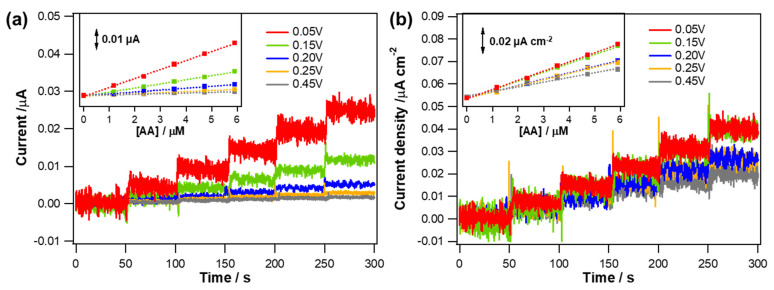
(**a**) Typical dynamic current response curves of the various Au electrodes electrodeposited at different deposition potentials (0.05 V to 0.45 V) to AA concentration changes in deaerated PBS solution (pH 7.4) measured at an applied potential of −0.05 V (vs. Ag/AgCl). (**b**) The current data in (**a**) were normalized to the corresponding electrode ESAs. Insets show the corresponding calibration curves.

**Figure 6:**
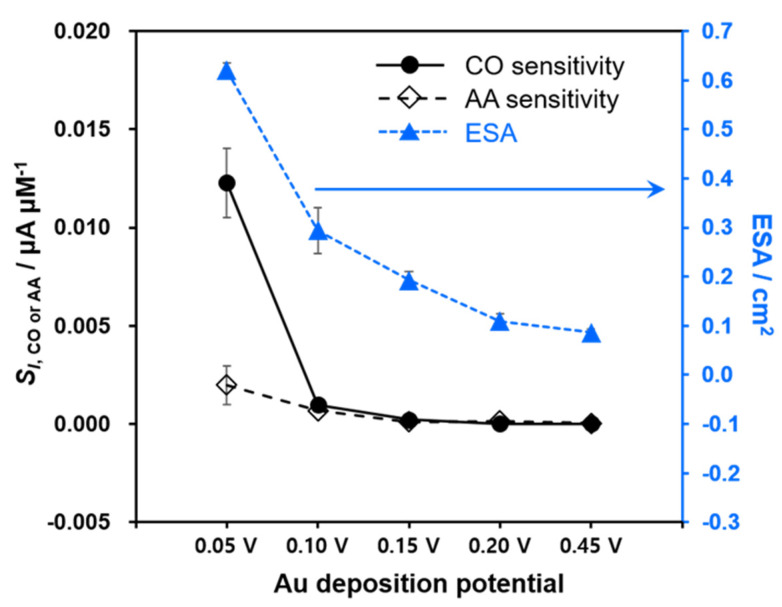
The current sensitivities of Au deposits to CO and AA along with the corresponding ESAs depending on the deposition potential (*n* = 5).

The current and current density responses of these Au deposits to typical biological interfering substances were also observed at the same applied potential of −0.05 V (Figure 4). The electrode current signals were barely changed in response to the addition of 5 µM AP, GABA and NO_2_^−^. However, the injection of 5 µM AA induced the anodic current increases for all the Au deposits indicating the catalytic activity of Au for AA oxidation as previously reported [32,33,34]. To investigate the behaviors responding to AA in more detail, the electrode currents were monitored with several increments of AA concentration. As shown in Figure 5a, the measured current was linearly proportional to AA concentration while the current sensitivity (*S_I_*_,AA_) was different depending on the Au deposits. The Au layers exhibited higher *S_I_*_,AA_ as the deposition potential decreased from 0.45 V to 0.05 V. The current density sensitivity (*S_J_*_,AA_) obtained after the normalization were quite similar to one another among the various Au deposits in contrast to the case of *S_J_*_,CO_. Figure 6 shows the tendency of *S_I_*_,CO_, *S_I_*_,AA_ and ESA as a function of Au deposition potential. In fact, *S_I_*_,AA_ increased gradually with lowering the deposition potential throughout all the different deposits that could be ascribed to the enlarged ESA. Likewise, *S_I_*_,CO_ also increased rather gradually as the deposition potential became less positive from 0.45 V to 0.10 V. However, a further decrease in the deposition potential down to 0.05 V enhanced *S_I_*_,CO_ greatly, exceeding the pattern of *S_I_*_,AA_. Indeed, the Au layer deposited at 0.05 V eventually became much more sensitive to CO than AA, while all the other Au deposits exhibited similar levels of *S_I_*_,CO_ and *S_I_*_,AA_. This observation reasonably suggests that the great hydrophobicity of Au deposit attained at 0.05 V provides the selectivity to CO over anionic AA regardless of the high electroactivity of Au for AA oxidation. CO, a hydrophobic neutral gas molecule, seemingly has better access to the inner part of the most hydrophobic Au deposit and utilizes a larger surface area of Au for its oxidation.

The amperometric selectivity coefficients ($\log K_{\mathrm{CO},\;x}^{\mathrm{amp}}$) over the interfering species (x) were calculated for various Au deposits as follows:

(2) $$\log K_{\mathrm{CO},x}^{\mathrm{amp}}=\;\log\left(\frac{S_{I,x}}{S_{I,\mathrm{CO}}}\right)$$

where *S_I_*_,x_ is the current sensitivity to x species (AA, AP, GABA and NO_2_^−^).

In general, CO selectivity over all the tested interfering species was improved as the Au deposit became more hydrophobic. Thus, the Au deposited at 0.05 V showed the most negative $\log K_{\mathrm{CO},x}^{\mathrm{amp}}$ values; in other words, the highest CO selectivity among the as-prepared electrodes (Table 1). In fact, the Au electrodes produced at deposition potentials of 0.45 and 0.25 V were more sensitive to AA than CO (i.e., $\log K_{\mathrm{CO},\mathrm{AA}}^{\mathrm{amp}}$ > 0), indicating the inherent high electroactivity of Au for AA oxidation. On the other hand, the Au deposits formed at 0.20 V, 0.15 V and 0.05 V had negative values of $\log K_{\mathrm{CO},x}^{\mathrm{amp}}$ which became more negative with lowering deposition potential. The main finding of this study is that the selectivity to CO over interfering substances is possibly enhanced by reinforcing the hydrophobicity of the Au electrode surface, which was easily acquired by controlling the deposition potentials without additional modifications.

Our group has previously reported that an electrodeposited Pt layer with a spiky nanostructure and a great hydrophobicity showed enhanced sensitivity in amperometric sensing of NO [22]. In current work, that Pt deposit was compared with the most hydrophobic Au deposit in terms of the CO sensing performance. The Pt layer was electrodeposited in a precursor solution containing 5 mM K_2_PtCl_4_ and 0.1 M H_2_SO_4_ using coulometry at −0.2 V (vs. Ag/AgCl) with a deposition charge of 0.08 C as described previously [22]. Figure S4 shows dynamic current response curves and the corresponding calibration curves of the Au and Pt deposits to concentration changes of CO and NO (0~18.2 µM for CO, 0~0.53 µM for NO). A wider concentration range was selected for a calibration curve of CO due to the higher physiological CO levels than NO [35]. Because of the similarities such as size and polarity of these two electroactive gases, different potentials were applied for selective amperometric sensing of each gas molecule. In fact, the optimized applied potentials (*E*_app_) of Au and Pt deposits for CO sensing were −0.05 V and +0.4 V vs. Ag/AgCl, respectively. As seen in Figure S4a, Au electrode at *E*_app_ = −0.05 V showed anodic current increase proportional to only CO concentration and barely responded to NO concentration change, confirming the selectivity to CO over NO. In contrast, both the CO oxidation current and NO reduction current were observed at Pt deposited electrode applied with the same potential of −0.05 V (Figure 3b). In addition, the response time to CO oxidation was very slow at Pt deposited electrode and rarely responds to low concentrations (0–3.58 μM), causing poor linearity (R^2^ = 0.94) within a tested concentration range (0–18.2 µM). Au electrode at *E*_app_ = +0.4 V also responded almost exclusively to CO. However, the CO selectivity over NO was slightly diminished from −3.04 to −1.06, because the sensitivity toward CO increased a little but that for NO also increased compared to the case of *E*_app_ = −0.05 V (Figure S4c). The current of Pt electrode at *E*_app_ = +0.4 V appeared to increase exclusively by CO oxidation, but the current at the moments of injecting a NO standard solution was very unstable (Figure S4d). Only a very small potential change switches between the oxidation and the reduction of NO on Pt deposit and therefore it seems to be hard to find a potential not allowing redox reaction of NO. Besides, the selectivity of Pt electrode to CO over NO was not as good as that of Au electrode at each respective optimized potential. The $\log K_{\mathrm{CO},\mathrm{NO}}^{\mathrm{amp}}$ values were −3.04 and −1.28 for Au (*E*_app_ = −0.05 V) and Pt (*E*_app_ = +0.4 V) electrodes, respectively.

The CO selectivity of Pt electrode over other biological interferents was also investigated and compared with that of Au electrode in Table S1. Compared to Au deposit, Pt showed less negative $\log K_{\mathrm{CO},x}^{\mathrm{amp}}$ values regarding all the tested interferents, indicating its lower CO selectivity. Conclusively, Au deposit having a very sharp morphology is feasible to sense CO sensitively and selectively which is attributed to the great hydrophobicity of deposited Au surface and the intrinsic nature of Au itself. 

## 4. Conclusions

Diverse Au nanostructures were electrodeposited successfully by controlling the deposition potential. Reducing the overpotential for Au precursor reduction induced the smaller cathodic current or deposition rate, and therefore the less sharp morphology was obtained. In fact, the actual structures of Au deposits were changed from sharp pine tree-like shapes to a smooth round shape with increasing the deposition potential from 0.05 V to 0.45 V. Depending on their morphologies, the hydrophobicity of the Au structures was differed; the deposit with a sharper morphology showed a more hydrophobic characteristic. As the Au surface became more hydrophobic, higher sensitivity to CO oxidation was obtained even after the current normalization to ESA. CO selectivity over common biological interferents (AA, AP, GABA and NO_2_^−^) was also improved as the Au deposit became more hydrophobic. This is possibly ascribed to that neutral gaseous CO can utilize larger Au surface for the oxidation in the case of more hydrophobic Au layer. In fact, the most hydrophobic Au formed with 0.05 V deposition showed better sensitivity to CO than AA despite the high activity of Au for AA oxidation. In addition, the most hydrophobic Au showed higher CO selectivity over NO and other biological interferents compared with the most hydrophobic Pt. This study suggests that the wettability control of the Au nanostructures can strengthen the sensitivity and selectivity in amperometric CO measurements.

## Figures and Tables

**Table 1:** Selectivity coefficients ($\log K_{\mathrm{CO},x}^{\mathrm{amp}}$ ) of the Au electrodes deposited with various deposition potentials to CO over typical biological interferents (x).

x	Deposition Potential				
	0.05 V	0.15 V	0.20 V	0.25 V	0.45 V
AA	−0.714	−0.294	−0.141	0.517	0.942
AP	−3.944	−2.512	−2.511	−2.146	−1.602
GABA	−3.768	−2.335	−1.968	−1.845	−1.757
NO_2_^−^	−3.665	−3.233	−2.513	−2.146	−1.601

## Supplementary Materials

The following are available online at https://www.mdpi.com/article/10.3390/bios11090334/s1, Figure S1: Potentiostatic electrodeposition curves of the Au electrodes at various deposition potentials, Figure S2: SEM image of the Au nanostructure using a precursor solution containing 7 mM HAuCl_4_ aqueous solution without Pb(CH_3_COO)_2_, Figure S3: Water droplets on the various Au surfaces, Figure S4: Dynamic current response curves of Au and Pt deposited electrodes to the concentration changes of CO and NO, Table S1: Selectivity coefficients of the Au and Pt electrodes for CO over typical biological interferents.
