# Supplementary material for: Amperometric Sensing of Carbon Monoxide: Improved Sensitivity and Selectivity via Nanostructure-Controlled Electrodeposition of Gold
Source: Biosensors (Basel). 2021 Sep 13;11(9):334. doi: 10.3390/bios11090334 (PMC8468895; doi:10.3390/bios11090334)
Supplement: Supplementary file 1 [file biosensors-11-00334-s001.zip › biosensors-1356248-supplementary.pdf]

*Supplementary Materials*

# Amperometric Sensing of Carbon Monoxide: Improved Sensitivity and Selectivity via Nanostructure-Controlled Electrodeposition of Gold

**Taehui Kwon †, Hee Young Mun †, Sunghwa Seo †, Areum Yu, Chongmok Lee and Youngmi Lee***

Department of Chemistry & Nanoscience, Ewha Womans University, Seoul 03760, Korea
†Authors contribute this work equally.
* Correspondence:

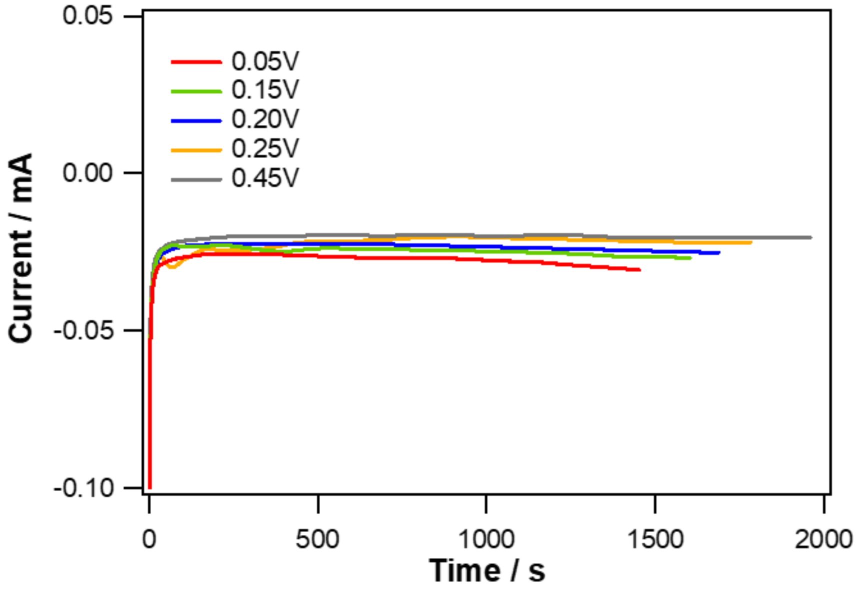


**Figure S1.** Potentiostatic electrodeposition curves of the Au electrodes at various deposition potentials (0.05 V to 0.45 V).

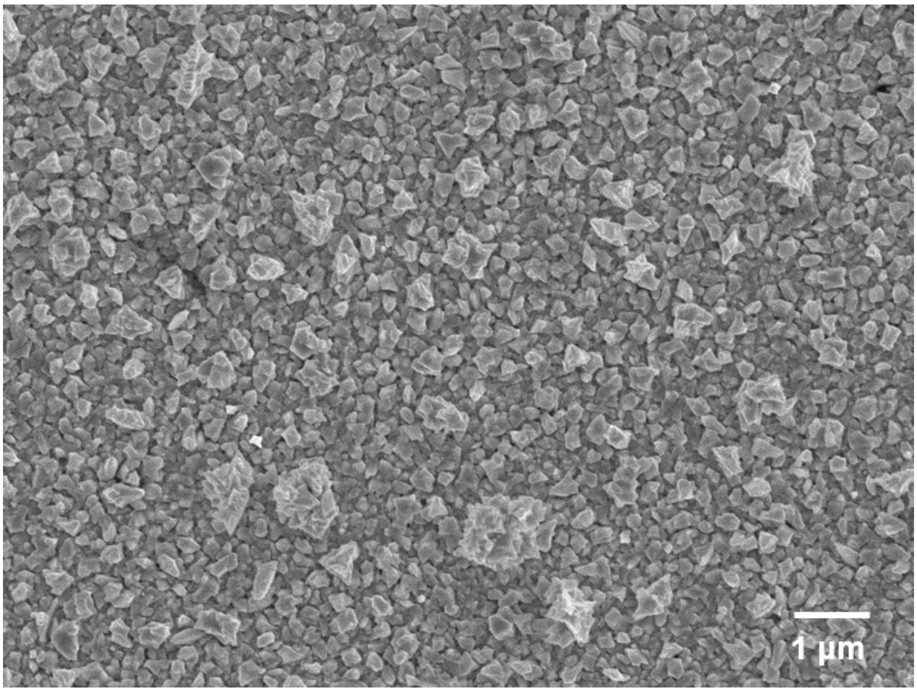


**Figure S2.** SEM image of the Au nanostructure electrodeposited at 0.05 V using a precursor solution containing 7 mM $HAuCl_4$ aqueous solution without $Pb(CH_3COO)_2$.

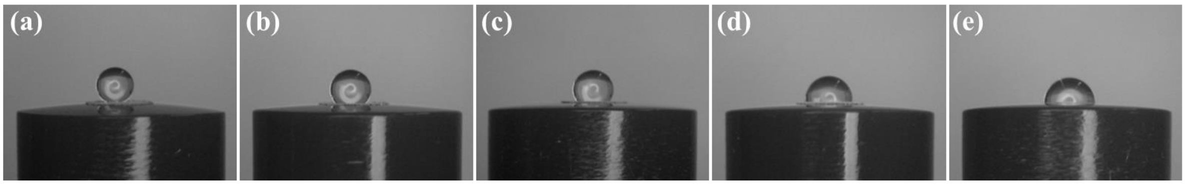


**Figure S3.** Water droplets (1 μL) on the Au surfaces deposited with deposition potentials of (a) 0.05 V, (b) 0.15 V, (c) 0.20 V, (d) 0.25 V, and (e) 0.45 V.

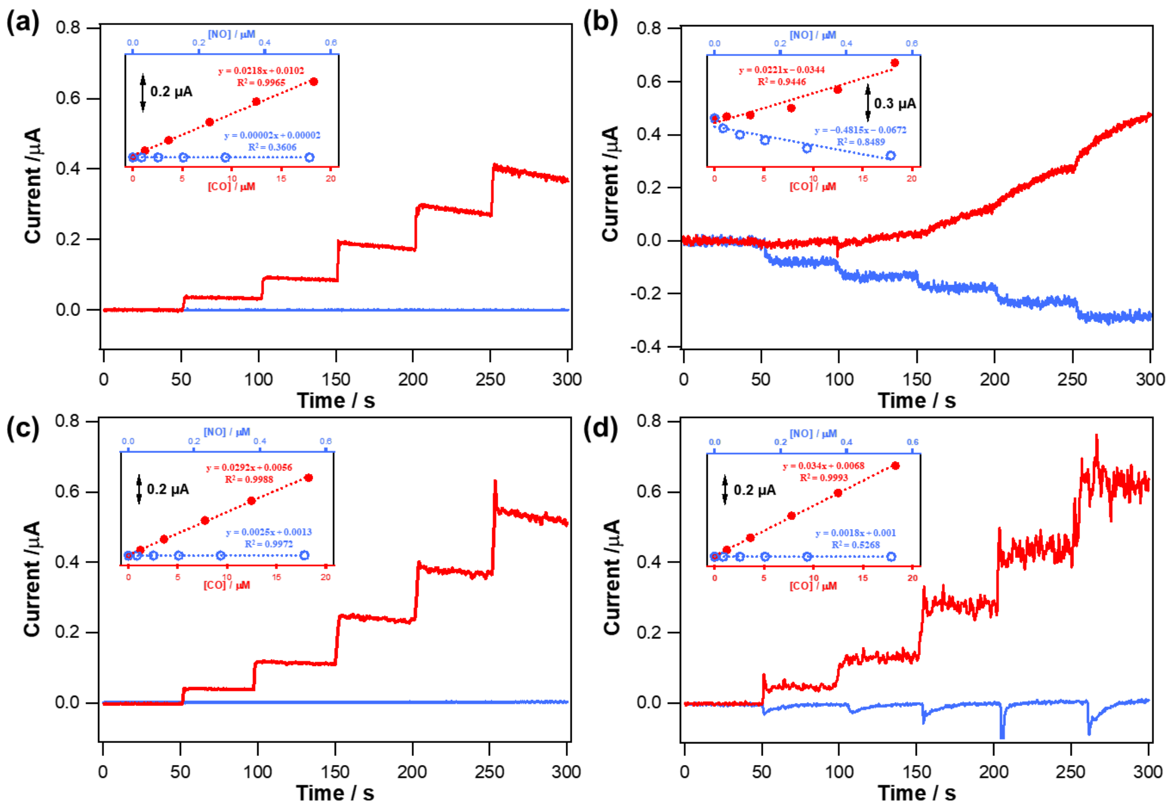


**Figure S4.** Dynamic current response curves of (a,c) Au and (b,d) Pt deposited electrodes to the concentration changes of CO (red line) and NO (blue line) in deaerated PBS solution (pH 7.4). $E_{app}$ = (a,b) −0.05 V and (c,d) +0.4 V vs Ag/AgCl. Insets show the corresponding calibration curves (red filled circle, CO; blue open circle, NO). The concentration was increased upto 18.2 μM and 0.53 μM for CO and NO, respectively.

**Table S1.** Selectivity coefficients ($\log K_{CO,x}^{amp}$) of the Au and Pt electrodes for CO over typical biological interferents (x).

| x | Electrode | |
|---|---|---|
| | **Au deposit** | **Pt deposit** |
| AA | −0.714 | −0.405 |
| AP | −3.944 | −0.106 |
| GABA | −3.768 | −0.122 |
| $NO_2^-$ | −3.665 | 0.336 |
